# Performance of OncoE6 cervical test with collection methods enabling self-sampling

**DOI:** 10.1186/s12905-018-0559-3

**Published:** 2018-05-21

**Authors:** Amrei Krings, Anna M. Dückelmann, Lutz Moser, Johannes Gollrad, Maarten Wiegerinck, Johannes Schweizer, Andreas M. Kaufmann

**Affiliations:** 1 Clinic for Gynecology CCM/CBF, Charité-Universitätsmedizin Berlin, corporate member of Freie Universität Berlin, Humboldt-Universität zu Berlin, and Berlin Institute of Health, Berlin, Germany; 2 Department of Radiooncology, CBF, Charité-Universitätsmedizin Berlin, corporate member of Freie Universität Berlin, Humboldt-Universität zu Berlin, and Berlin Institute of Health, Berlin, Germany; 3 Mysample Device Diagnostics B.V, Amsterdam, The Netherlands; 4 Arbor Vita Corporation, Fremont, CA USA; 5 Gynäkologische Tumorimmunologie, Charité-Universitätsmedizin Berlin, Campus Benjamin Franklin, Hindenburgdamm 30 Room # 4503, 12200 Berlin, Germany

**Keywords:** Low-resource, Self-collection, Oncoprotein testing, Cervical cancer screening, HPV testing

## Abstract

**Background:**

The paradigm shift from cytological screening to Human Papillomavirus (HPV)-based screening for cervical cancer allows the introduction of new technologies in sample collection and diagnostics. The Onco***E6***™ Cervical Test (OncoE6 Test) is a rapid, easy-to-use lateral flow method detecting HPV16/18 E6 oncoproteins that has proven to detect high-grade cervical lesions with high specificity. If compatible with self-collection samples, this technology might allow for decentralized screening of hard-to-reach populations.

**Methods:**

For technical validation, cervicovaginal lavages were collected from 20 patients with confirmed HPV16+ or HPV18+ invasive cervical cancer. Cervical smears were collected by polyester-tipped swabs and cytobrushes. All samples were applied to the OncoE6 Test and cytobrush samples additionally genotyped.

**Results:**

Lavage, swab, and cytobrush revealed concordant outcome in 18/20 samples. HPV types corresponded with the HPV genotyping by GP5+/6+ PCR analyses. Due to a rare mutation found in the E6 antibody binding site one sample was not detected, another sample had very low cellularity.

**Conclusions:**

Overall, vaginal lavages are technically adequate for the OncoE6 Test. Combining self-sampling with oncoprotein rapid testing to detect women with highest risk for severe dysplasia or cancer may allow for secondary cancer prevention in settings where other screening modalities were unsuccessful to date.

## Background

Despite successes in reduction of cervical cancer related mortality by the introduction of screening programs in developed regions, the worldwide cervical cancer incidence remains high, especially in developing countries. The World Health Organization (WHO) estimates more than 550.000 new cases annually [1]. Cervical cancer is a relatively rare consequence of high-risk human papillomaviruses (hr-HPV) infection and the WHO has classified 12 h-HPV genotypes detectable in > 95% of invasive cervical squamous carcinoma [2, 3]. While hr-HPV infection has a relatively high prevalence of 5–20% in many regions, > 20% can be observed in regions of high HIV prevalence and in women < 25 years old [1]. The HPV types 16 and 18 account for more than 70% of cervical cancers [4].

The association of cervical cancer with hr-HPV (virtually 100% of cervical squamous cell carcinomas are caused by HPV) has motivated the development of molecular screening tests detecting presence of HPV, and WHO recommends the introduction of such screening tools [5]. Generally, HPV tests are characterized by high sensitivity but suffer from low specificity for true disease due to many infections resolving spontaneously or not leading to cervical cancer (HPV infection rarely result in cervical cancer) [6].

A necessary pathogenic event during cervical cancer carcinogenesis is the upregulation of HPV encoded oncoprotein expression. The maintained expression of HPV oncoproteins E6 and E7 is a prerequisite for invasive cervical cancer to develop, while levels of the E6 and E7 oncoproteins and the corresponding mRNAs are at a very low level in non-transforming HPV infections and low grade dysplasia [7, 8]. These findings motivated the development of the Onco***E6***™ Cervical Test (OncoE6 Test), a technology directly detecting elevated levels of the E6 oncoprotein of HPV types 16 and 18 [9]. The OncoE6 Test relies on genotype-specific mouse monoclonal antibodies (mAbs) to HPV 16 E6 and HPV 18 E6 oncoproteins; these mAbs are used in the format of a lateral flow assay (strip test) of high robustness [9].

The OncoE6 lateral flow assay was validated in numerous clinical studies, and initially only physician collected specimens were used in those studies. For example, in a large (~ 7500 subjects enrolled) screening study conducted in several rural areas in China, the OncoE6 Test had the highest specificity for detection of CIN3 and cervical cancer among all tests involved (compared to *care*HPV and VIA) at 98.9%; the sensitivity of the OncoE6 Test for CIN3+ was 53.5% for all lesions detected and 70.3% when stratified for lesions positive for the HPV types 16/18/45. In this study, the OncoE6 Test had by far the highest positive predictive value (PPV) for CIN3 of 40.8%, while the PPV of HPV testing and VIA was < 10%; the negative predictive value was 99.4% [10].

In low resource settings, tests with high specificity that are also compatible with self-sampling would be advantageous as they facilitate cervical cancer screening of women in remote locations and of women who are hesitant to present to physicians, while reducing unnecessary referrals [11, 12]. We therefore investigated compatibility of the OncoE6 Test with specimens collected by self-sampling. We chose to use cervicovaginal lavages that had previously been shown to be compatible with HPV genotyping [11, 13, 14]; we compared OncoE6 Test performance on self-sampling device collected specimens with physician collected swabs and the cervical brush samples. For this truly technical comparison we sampled from women with histologically confirmed cervical cancers caused by HPV genotypes 16 or 18, and we tested the different sampling methods with regard to the Onco E6 Test, comparing the results to the molecular HPV genotyping performed on the cytobrush samples.

## Methods

### Patient and samples

Patients were eligible for the study if they presented with HPV 16 or 18 positive invasive cervical cancer, if they have not had a history of conisation or other treatments for cervical cancer, if they were not pregnant and gave their written consent to participate in the study. Ethical approval for this study was granted by Charité Universitätsmedizin Berlin ethical review board (EA1/168/13).

From each patient three different samples were collected in the following order: a vaginal lavage sample (1) taken with the self-sampling device Delphi Screener (Rovers Medical Devices, Oss, Netherlands), a dry sample (2) collected with a sterile polyester tipped swab (#25–806 1PD, Puritan Medical Products) and a cytobrush sample (3) that was applied to PreservCyt storage media (ThinPrep, Hologic Inc., Marlborough, MA, USA). Before the swab and cytobrush sampling, a speculum was inserted and these two samples were collected from the cervix or, if visible, directly by touching the tumour. All three samples were collected by a physician. The samples were stored at − 20 °C until processing.

### OncoE6 test

All three samples were applied to the Onco***E6***™ Cervical Test (Arbor Vita Corporation, Fremont, CA, USA) following the manufacturer’s instructions. The lavage sample (1) was centrifuged and the whole cell pellet re-suspended in lysis buffer. The entire lavage sample was used for the OncoE6 Test to assure highest yield of cells and hence the best possible results. The swab sample (2), was transferred into a tube for lysis without any further preparation, according to the standard protocol provided for the OncoE6 Test. Of the cytobrush sample (3) in PreservCyt storage media, 5 out of 20 ml of cell suspension were centrifuged and the cell pellet was re-suspended in lysis buffer and subsequently applied to the OncoE6 Test. The protocol provided by Arbor Vita Corporation for PreservCyt samples recommends as little as 2 ml cell suspension. We used 5 ml to assure the expected amount of cells would be sufficient.

The OncoE6 Test results for all three sample types were compared to the HPV genotyping outcome, and subsequently the lavage and cytobrush sample results were compared to the swab-based test result.

### HPV DNA-based testing

HPV genotyping was performed using the cytobrush sample (3) kept in storage media; 1 ml of this cell suspension was used. DNA was extracted using the QIAmp DNA mini kit (Qiagen, Hilden, Germany) and following the manufacturer’s instructions. The extracted DNA was submitted to genotyping using the BSGP5+/6+ PCR approach followed by a Luminex-based readout [15]. The outcome constituted the reference result for HPV genotype status of the patient.

### Additional analysis

For all cytobrush samples DNA concentrations were measured using the NanoDrop 2000 (Thermo Scientific, Wilmington, USA) device. It was not possible to measure DNA concentration from the lavage and swab sample, as those were fully used for the OncoE6 test.

One sample (#5) was applied to DNA sequencing of the E6/E7 gene region in the HPV genome (Eurofins Genomics GmbH, Ebersberg, Germany). Primers encompassed the E6 region of HPV 16 (NCBI accession #: NC_001526) and had the following nucleotide sequences:

Forward: TTGAACCGAAACCGGTTAGT

Reverse: AGATCAGTTGTCTCTGGTTGC

## Results

Complete sample sets (a “sample set” consists of: lavage, swab, and cytobrush) were obtained from 20 patients with histologically confirmed invasive cervical cancers; subjects had been pre-selected for infections with HPV types 16 and/or HPV 18; the patients presented at the Clinic for Gynaecology or Radiology, Charité University Hospital in Berlin for treatment. The median patient age was 45 years, ranging from 26 years to 64 years. The patients came to the clinic for surgery and/or radiochemotherapy, at which occasion the samples were collected. None of the patients had previously received treatment for cervical cancer (some patients had to be excluded from the analysis because their medical record revealed prior treatment for cervical cancer).

### HPV genotyping results

The genotyping results obtained at the time of sample collection confirmed that 15 out of the 20 cervical cancer patients were positive for HPV 16 and the remaining 5 patients for HPV 18. Three patients each were also positive for additional high-risk HPV types albeit at lower virus load or with a borderline positive result. None was positive for HPV 16 and HPV 18 simultaneously.

### OncoE6 test results

The outcome of the OncoE6 Test for lavage, swab and cytobrush samples was highly consistent (see Table 1). E6 oncoprotein positive types by the OncoE6 Test also corresponded to the HPV types 16 or 18 as detected by BSGP5+/6+ PCR genotyping from the cytobrush reference sample. One sample (#14) had a very faint double positive result for HPV 18 and 16 (HPV16 weak) in the lavage sample, and two sample sets showed partially discordant results, warranting further examination as described below.

**Table 1:** HPV Testing results by sampling method (lavage, swab & cytobrush)

Sample number	DNA Concentration cytobrush sample (ng/μl)	HPV genotyping result from cytobrush (MFI values)		OncoE6 Test result		
				Lavage	Swab	Cytobrush
1	208,9	18 39	(128) (61)	18	18	18
2	30,4	16 33 52	(2020) (54) (231,5)	16	16	16
3	10,3	16	(375)	16	16	16
4	41,3	18	(38)	18 (weak)	18	18
5	34,0	16	(713)	neg	neg	neg
6	68,8	16	(1025,5)	16	16	16
7	27,3	18	(34)	18	18	18
8	63,1	16	(1698)	16	16	16
9	2,8	16	(867)	16 (weak)	16	16
10	33,7	16 33bl^a^ 35bl^a^ 39bl^a^	(4985) (5) (6) (5)	16	16	16
11	54,8	16 39	(4203) (6)	16	16	16
12	Not available	16	(2799,5)	16	16	16
13	Not available	16	(1008,5)	16	16	16
14	Not available	18 56	(112) (14)	18, 16 (weak)	18	18
15	6,0	16 59bl^a^	(19) (16)	neg	16	neg
16	132,5	16	(1700)	16	16	16
17	71,7	16	(2969,5)	16	16	16
18	6,0	16	(2317)	16	16	16
19	68,7	18 43	(134) (8,5)	18	18	18
20	22,2	16	(2031)	16	16	16

^a^ borderline result

### Verification of discordant assay results

Sample set # 5 (see Table 1) repeatedly tested negative for the OncoE6 Test with all three sample types (Delphi Screener lavage, Swab, and Cytobrush), while the L1-based BSGP5+/6+ PCR MPG HPV typing clearly indicated presence of HPV 16. This sample was therefore examined for mutations in the HPV 16 E6 DNA sequence, hypothesizing that such mutations might alter the E6 oncoprotein binding to the anti-E6 specific monoclonal antibodies (mAb) applied in the OncoE6 Test. The primers chosen encompassed the sequence coding for the epitope recognized by the respective mAb included in the OncoE6 Test. The sequence analysis revealed a single nucleotide exchange (point mutation) at N120 from adenine to guanine, resulting in an altered E6 protein amino acid sequence in position 6 (Gln to Arg). Another point mutation was detected at N350 from thymine to guanine, coding for position 90 of the E6 protein amino acid sequence and resulting in a change from Leu to Val. We hypothesize that the mutation in position 6 reduced binding strength of the epitope to the anti-E6 mAb used in the OncoE6 Test, thus resulting in a false negative outcome.

The second discordant sample set (# 15), tested negative for HPV 16 with the Delphi Screener lavage sample and the cytobrush sample, but tested positive for HPV 16 from the swab sample (the latter in concordance with the control genotyping). Possible reasons for these false negative results could be insufficient cellularity in the respective samples. To judge cellularity of the false negative sample we compared DNA content of the cytobrush samples by NanoDrop measurements as an indirect measure of possible cellularity problems. A low DNA concentration was seen in the cytobrush collected sample with only 6 ng/μl while positive samples had a mean content of 51.9 ng/μl (range 2.8 to 208.9). Sample # 9 and #18 had low DNA concentrations in the cytobrush samples as well with 2.8 and 6 ng/μl, respectively, while the OncoE6 results for these samples were positive.

Based on these test results the sensitivity to detect HPV 16 or 18 positive cervical cancers by the OncoE6 Test with the Delphi Screener lavage and the cytobrush sample in PreservCyt media was determined at 90% and for the swab sample at 95%. After exclusion of sample set #5 due to the E6 epitope mutation described above, sensitivity can be adjusted to 95%. For the swab sample, sensitivity was 100% (Table 2). Specificity cannot be calculated, as none HPV 16 or 18 negative patients were included in this initial study enrolment.

**Table 2:** OncoE6 Test Positivity rates by sampling method

Samples tested (*n*=19^a^)	Lavage	Swab	Cytobrush
OncoE6 pos.	18 (95%)	19 (100%)	18 (95%)
OncoE6 neg.	1 (5%)	0 (0%)	1 (5%)

^a^ One sample excluded due to E6 epitope mutation

## Discussion

The shift from cytology-based cervical cancer screening to molecular-based screening opens the path for self-sampling in conjunction with nucleic acid based HPV tests and potentially also with HPV oncoprotein tests [12]. The Delphi Screener is a self-sampling device that is easy to use, well accepted by the users and that results in sufficient material for HPV DNA testing [11, 14, 16]. This device, however, had never been validated in conjunction with the OncoE6 Test, for which most studies up to date were done using swab samples. Here, we performed a technical validation study using the Delphi screener in conjunction with the OncoE6 Test. For this, we selected 20 patients with HPV 16 or HPV 18 positive histology confirmed cervical cancers. Three different sample types were obtained (Delphi lavage, swab, cytobrush) and performance of OncoE6 Test performance was determined for these sample types, using histology as the gold standard for pathology and the L1-based BSGP5+/6+ PCR Multiplexed Genotyping with Luminex readout as the standard for HPV status. The result suggests high sensitivity for detection of cervical cancer by the OncoE6 Test using all three sample types, the self-sampling device generated specimens, the swab samples and also the cytobrush liquid media-based samples.

Two of the 20 samples revealed discordant outcome resulting in a sensitivity of 90% for the self-sampling device, however, this may not be applicable with regard to cervical cancer screening by the OncoE6 Test in a population wide setting, as substantiated by the outcome of our further analysis.

One sample set (# 5) was negative in OncoE6 testing for all three sampling methods, despite a strong positive signal for HPV 16 in the L1-based genotyping assay. Sequencing of the E6-specific epitope coding gene segment revealed a non-silent mutation within the E6 gene sequence, suggesting that the resulting epitope has reduced binding strength to the anti E6 mAb used in the OncoE6 Test. Review of the literature for description of E6 specific mutations revealed several studies from various countries (e.g. Congo, Morocco, Romania, China). The mutation we found in our sample set # 5, however, was not described in any of the studies [17–25]. We therefore conclude that this mutation represents a very low frequency mutation and will thus not influence the clinical performance of the OncoE6 Test in the general population, since it is highly unlikely to find this same mutation in relevant numbers elsewhere. Future analyses of discrepant screening by OncoE6 Test results should include interrogation for this mutation.

A major objective of our study was to assess whether or not the Delphi Screener lavage sample self-sampling device is in principle technically compatible with the OncoE6 Test; we therefore focused on samples from HPV 16/18 positive histologically confirmed cervical cancer patients. Excluding the one inadequate sample from our analysis, sensitivity for the self-sampling device among cervical cancer patients selected for this technical validation is at 95% (18/19 samples correctly detected) for the detection of HPV 16 or 18 induced cervical cancers using the Delphi Screener lavage. It can be expected that such very rare mutations do not reduce sensitivity of the OncoE6 test in population-based screening significantly.

The second sample set (# 15) resulting in discrepant outcome showed negative results for the lavage sample as well as the cytobrush sample with the OncoE6 Test, while the result for the swab sample was positive for E6 oncoprotein. It was noticeable that HPV genotyping performed from the cytobrush sample in PreservCyt media showed very low signal strength with the semi-quantitative BSGP5+/6+ PCR with Luminex readout, suggesting the sample was very low on HPV positive cells. To investigate further, we measured the DNA concentration in the cytobrush samples and this sample had a DNA concentration of only 6 ng/μl. Potentially, only few cancer cells were collected, or the tumour was not directly accessible for sample collection, resulting in negative test results for the cytobrush and the lavage sample. It is possible, therefore, that the number of E6 expressing cells was too low, thus resulting in a negative OncoE6 Test. In contrast, sample #9 and #18 showed a low DNA concentration, suggesting low cellularity of the sample, but in this case it did not seem to have an impact on the genotyping results, which presented positive with high signal strength. As hypothesized previously, self-collected samples bear the risk of not containing enough biological material [26]. Also the location of the lesion, its size, viral load, and smear quality can vary between patients. Data from other studies indicate that even lesions that are too small to be seen in Colposcopy can yield enough E6 protein to be detected in the OncoE6 test [27]. In addition, samples collected with such devices are not specific to the lesion/tumour or the cervix but are cervico-vaginal samples that can contain various types of cells. This might affect the use of such samples in the OncoE6 Test. Since it is difficult to assess how many “correct” cells are collected with self-sampling devices, the impact on sensitivity and specificity of the test needs to be investigated further in population-based screening studies.

Weighing the advantages of wider population benefits due to extended screening possibilities against the drawbacks of a potentially limited sensitivity, we conclude that the Delphi Screener-based lavage sampling represents a very promising approach in conjunction with E6 oncoprotein testing. The undisputed advantages in ease of use and acceptance by the use of self-sampling in conjunction with the high positive predictive value and high specificity of disease detection, as demonstrated for the OncoE6 Test in population-based sampling studies [10], represent important features for effective cervical cancer screening. Elsewhere, the capacity of the OncoE6 Test to stratify risk and to predict >CIN3 at a time point where lesions are not yet visible has been described. In the study of Zhang et al., it was found that women who tested positive for E6 oncoprotein had a ten year cumulative incidence rate of 53.0% for >CIN3 [27]. This could potentially reduce over-referral and allow early and effective treatment of women at high risk, which is of great importance in low-middle income countries (LMICs) and in difficult to access populations.

Our study outcome warrants further investigation in larger population-based studies. The focus of our investigation was foremost to prove the technical feasibility of this approach, and we therefore concentrated on patients with invasive disease positive for HPV 16 or 18. This does not allow any conclusion on the sensitivity and specificity to be found among women with CIN2 or CIN3 lesions. Future studies should include a more typical screening population and thus allow determination of the clinical specificity of self-collection in conjunction with the OncoE6 Test and assess its usability as a screening test for early detection of lesions. The lower specificity of self-sampling devices has already been mentioned by Arbyn et al. [13] and should also be further investigated for this combination of self-sampling device and OncoE6 test.

Using self-sampling devices and E6 oncoprotein testing for cervical cancer screening would allow highly effective selection of women with a high-risk for cervical lesions. Reaching out by self-sampling to women who normally may not benefit from the screening activities or even avoid participation due to cultural barriers could enhance success of preventive strategies [28]. This is especially useful for decentralized cervical cancer screening in resource-constrained settings. A self-collection of lavage samples with the Delphi Screener allows sample collection without a doctor’s visit. Once a positive test result is obtained, the patient would be called to be referred to triage and treatment. Such approaches have been shown to motivate patients to consult a gynaecologist [28].

## Conclusions

This technical validation shows promising results for E6 oncoprotein testing from lavage samples obtained by a self-collection device, from swab samples and from cytobrush samples, yet further studies are be needed to investigate the feasibility in the general population. The feasibility of self-sampling in conjunction with the highly specific oncoprotein-based test may constitute an important element of cervical cancer secondary prevention in regions and resource constrained settings that could not be served in the past.

## Abbreviations

HPV: Human Papillomavirus
Hr-HPV: high-risk human papillomavirus
LMICs: low-middle income countries
mAbs: monoclonal antibodies
OncoE6 test: Onco***E6***™ Cervical Test
PPV: positive predictive value
WHO: World Health Organization
